# Experience sharing of a case of dual atrioventricular nodal non-reentrant tachycardia: Case report

**DOI:** 10.1097/MD.0000000000036401

**Published:** 2024-09-06

**Authors:** Yixuan Gao, Yan Wang, Ranzun Zhao, Du Yang, Lei Chen, Bei Shi

**Affiliations:** a Department of Cardiology, Affiliated Hospital of Zunyi Medical University, Zunyi, China.

**Keywords:** case report, DAVNNT, non-reentrant, supraventricular tachycardia

## Abstract

**Rationale:**

Tachycardia is a common arrhythmia in clinical practice, and its pathogenesis is mostly related to reentry. However, there are also a few tachycardia that are not related to reentry. Actively clarifying the pathogenesis of these non-reentry related tachycardia is of great significance for its treatment.

**Patient concerns:**

A 55-year-old female patient presented with recurrent palpitations with a fastest heart rate of 180 beats/minute 10 years ago.

**Diagnosis:**

Dual atrioventricular nodal non-reentrant tachycardia (DAVNNT).

**Interventions:**

DAVNNT can be cured by radiofrequency ablation of atrioventricular nodal slow path modification.

**Outcomes:**

The tachycardia has stopped.

**Conclusion:**

DAVNNT is a rare disease in clinical practice. Its characteristic is not reentration-related arrhythmias, but the phenomenon of increased heart rate caused by electrical conduction down the double pathway of atrioventricular nodal tract and subsequent pathway. Electrophysiological examination helps to clarify the diagnosis and pathogenesis, and catheter ablation can cure the disease.

## 1. Introduction

Tachycardia is a common tachyarrhythmia in clinical practice, and electrophysiological examination can clarify its pathogenesis and guide treatment. The pathogenesis of most tachycardia is related to reentry, but in a few cases, it is not related to reentry. This case is a final confirmed tachycardia unrelated to reentry.

**Figure 1.**
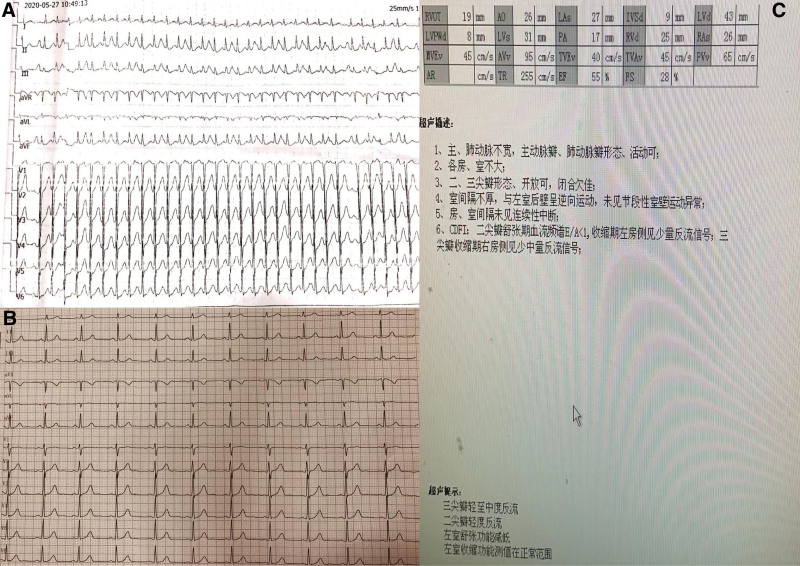
(A) ECG during past episodes showing paroxysmal tachycardia. (B) The ECG display of this hospitalization showing normal. (C) The results of cardiac ultrasound.

A 55-year-old Chinese female patient presented with recurrent palpitations with a fastest heart rate of 180 beats/minute 10 years ago without any obvious cause and was not treated. Two months ago, the patient’s palpitations recurred and worsened, and an electrocardiogram (ECG) at a community hospital showed tachycardia (Fig. 1A), and she was given oral treatment of “metoprolol tartrate 25 mg twice a day,” but her palpitations were not under satisfactory control, so she was admitted to our hospital. No abnormality was found on physical examination. ECG showed no abnormality (Fig. 1B), cardiac ultrasound: left atrial systolic diameter 27 mm, left ventricular diastolic diameter 43 mm, interventricular septum diameter 9 mm, ejection fraction 55% (Fig. 1C). No abnormalities were seen in routine blood, urine, stool, blood creatinine, or blood electrolytes. The patient was admitted to the hospital for treatment of suspected paroxysmal supraventricular tachycardia. The electrophysiology consultation recommended ablation, so cardiac electrophysiology was performed.

## 2. Perform intracardiac electrophysiological examination

**Figure 2.**
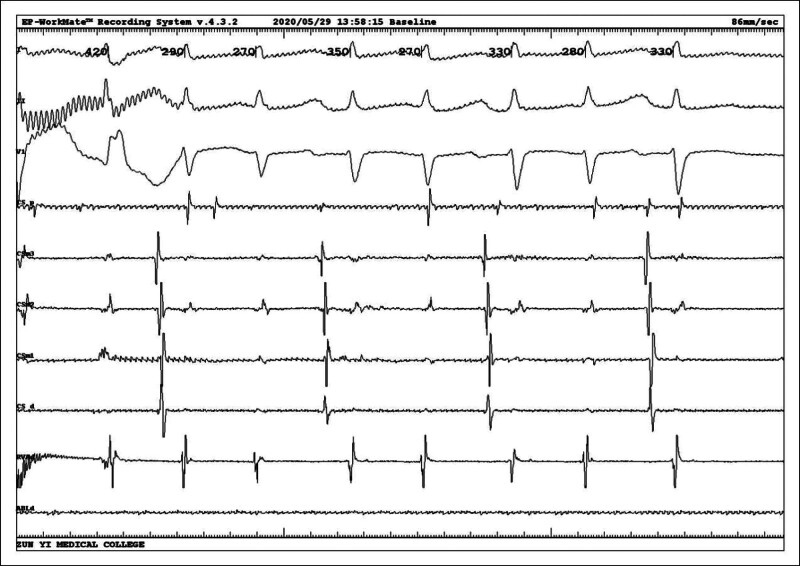
Intracavitary diagram at the beginning of the operation showing a continuous attack, AV was 1:2, and V-wave frequency was faster than A-wave.

**Figure 3.**
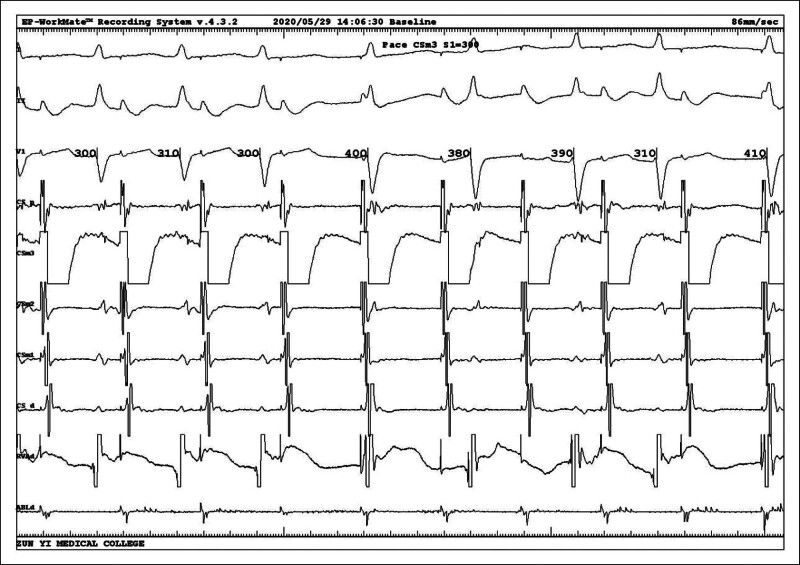
Coronary sinus middle 3 electrode (CSm3) S1S1 burst 300 ms Wenckebach block.

**Figure 4.**
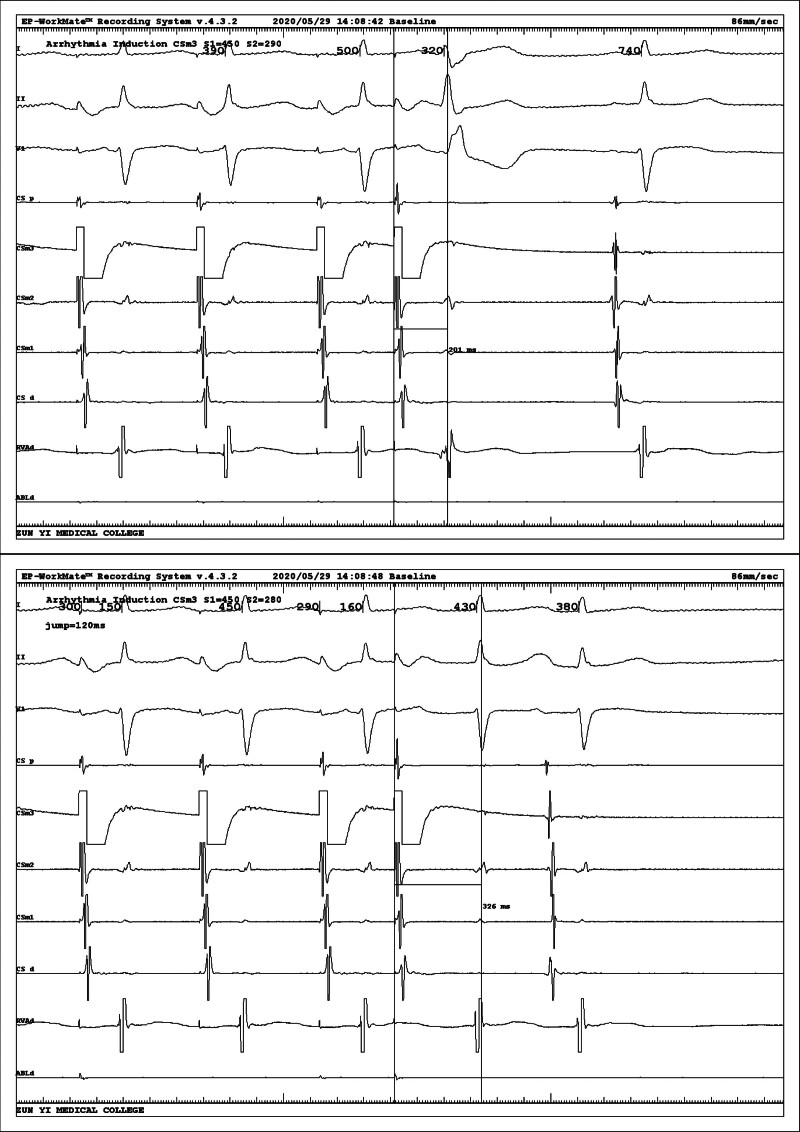
Coronary sinus middle 3 electrode (CSm3) S1S2 burst seeing jump 125 ms.

**Figure 5.**
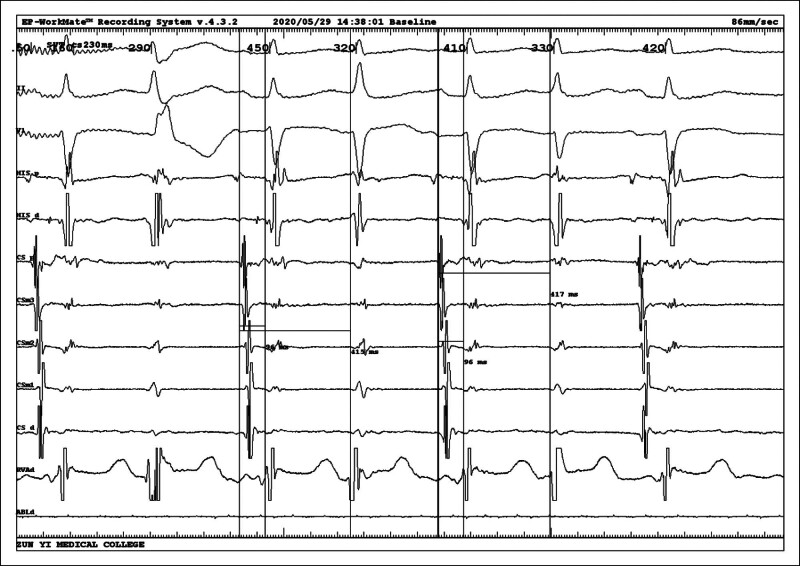
Placing his bundle electrode (HIS) electrodes showing AV presented a constant 1:2 conduction, HIS was present in both V-wave fronts, and the 2nd QRS wave combined with complete right bundle branch block (RBBB).

**Figure 6.**
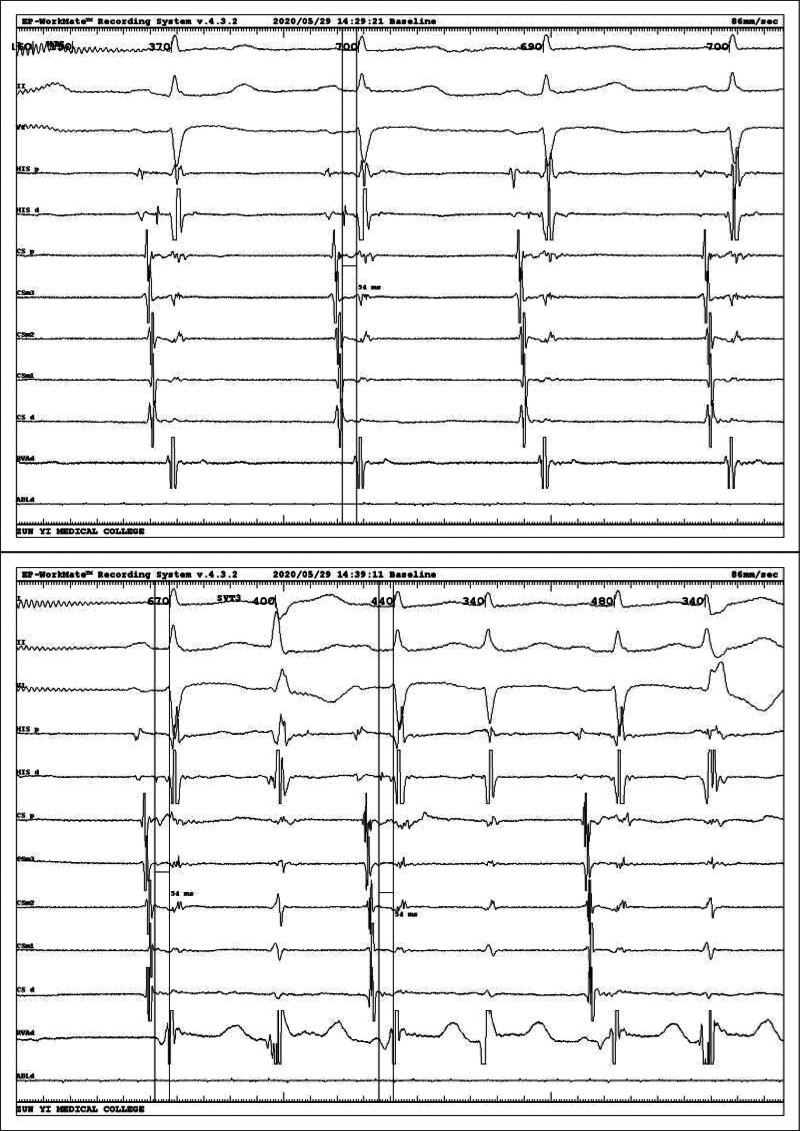
HV interval between attack and sinus rhythm was equal.

Intracardiac electrophysiological examination was arranged after admission to the hospital to exclude surgical contraindications, and coronary sinus electrodes and right ventricular electrodes (RV) were routinely placed. After the catheter was in place, it was found that the tachycardia showed a continuous attack (Fig. 2), A:V was 1:2, and ventricular wave (V-wave) frequency was faster than atrial wave (A-wave). Intracardiac electrophysiological examination was performed: (1) RV S1S1 burst showed no eccentric conduction, and RV S1S2 burst showed decreased conduction. (2) Coronary sinus middle 3 electrode S1S1 burst (Fig. 3) 300 ms Wenckebach block, coronary sinus middle 3 electrode S1S2 burst (Fig. 4) see jump 125 ms. (3) When his bundle electrode was placed (Fig. 5), A:V presented a constant 1:2 conduction, his bundle wave was present in both V-wave fronts, and HV interval was equal to HV interval in sinus condition (Fig. 6).

The relevant phenomena are summarized as follows: (1) A:V conduction is constant 1:2, his bundle wave potential is present in both V-wave fronts, and HV interphase is constant, indicating that both V waves are transmitted from A-wave, QRS presents a narrow QRS shape, regardless of ventricular tachycardia. (2) There was no eccentric conduction in RV burst, and there was a decrease, and bypass phenomenon could be basically excluded. (3) A jumping phenomenon was seen with coronary sinus burst, suggesting the presence of an AV node dual-pathway. (4) During the attack, the frequency of A-wave is not fast, does not conform to atrial tachycardia, and sometimes wide QRS is considered as the possibility of differential conduction.

**Figure 7.**
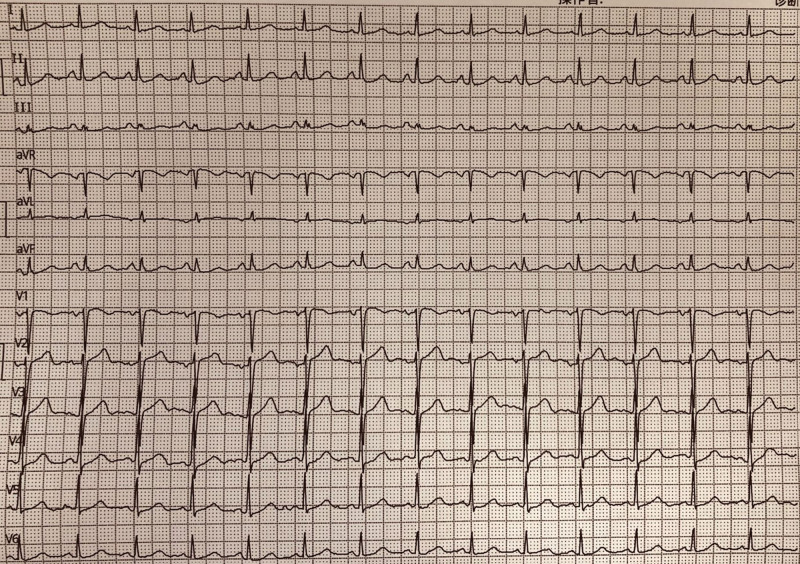
The ECG on the night of the surgery indicates normal.

**Figure 8.**
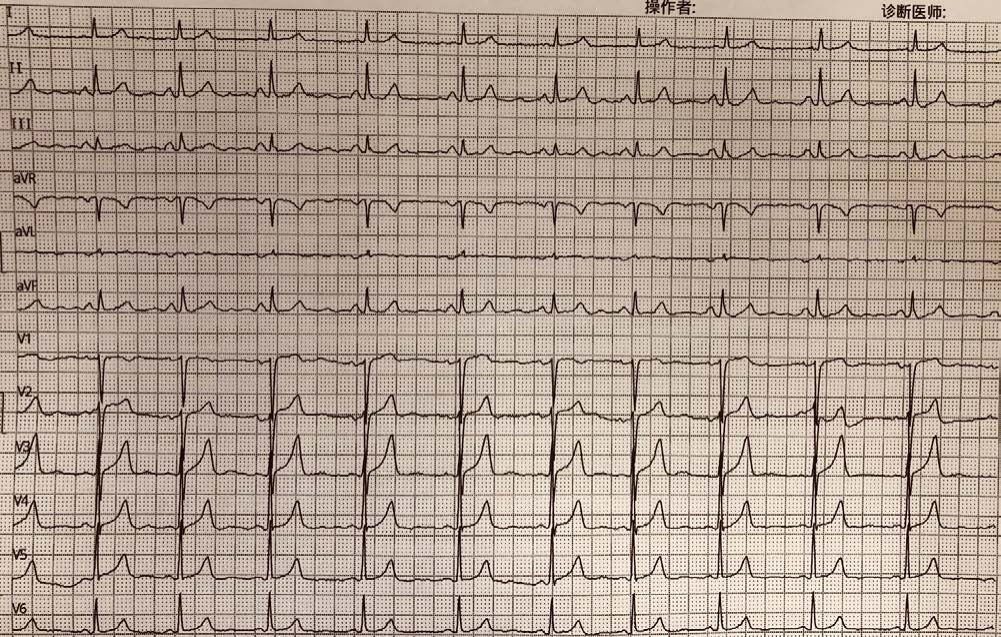
After 2 months of surgery, ECG showed normal results.

Taking into account the current information, tachycardia triggered by the AV node dual-pathway is considered to be highly probable, and the tachycardia was terminated after modified ablation of the slow pathway area was performed at 30 watt, 55 °C (during the ablation process, there is a phenomenon of alternating sinus rhythm and atrioventricular nodal rhythm, with a total ablation time of 180 seconds in the slow pathway area). Repeated postprocedural coronary sinus and RV burst and intravenous isoproterenol infusion failed to induce the tachycardia (Fig. 7). After 2 months of surgery, the patient returned to the hospital for re-examination and showed no symptoms of palpitations or tachycardia and the ECG was normal (Fig. 8).

## 3. Discussion

Dual atrioventricular nodal non-reentrant tachycardia (DAVNNT) is a relatively rare type of tachycardia,^[1]^ whose tachycardia episodes are associated with the AV node, which still has functionally different fast and slow pathways, but is different from atrioventricular reentrant tachycardia. However, unlike atrioventricular node reentrant tachycardia (AVNRT), the pathogenesis of DAVNNT is not intratrioventricular node reentry. Instead, atrial excitation is transmitted anteriorly along the fast and slow pathways, both of which excite the His and subsequent pathways, resulting in 2 ventricular beats, and presenting the characteristic “two birds with one stone” electrocardiogram. That is, 1-to-2 conduction or double ventricular excitation response.^[2–9]^

**Figure 9.**
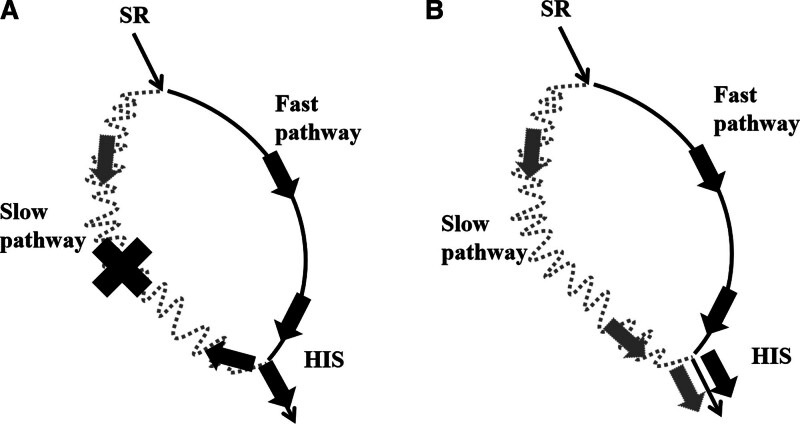
Schematic diagram of fast and slow path conduction of atrioventricular node under different conditions.^[]^

**Figure 10.**
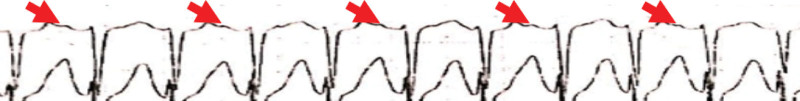
Review of V1 leads of surface electrocardiogram for out-of-hospital seizures, showing “Two birds with one stone.”

There are 2 paths of functional longitudinal separation in almost all people’s atrioventricular nodes, which are called fast path and slow path. The fast path has fast conduction speed and long refractory period, while the slow path has slow conduction speed and short refractory period. Under normal circumstances, when atrial excitation reaches the atrioventricular node, it will pass down the fast path and the slow path respectively. However, due to the fast conduction speed of the fast path, when it preferentially reaches the bundle of his, the excitation will not only continue to pass down the left and right fascicular branches, but also pass up and back the slow path. Because cardiomyocytes of the conduction system are conductive, the refractory period of the slow path is short and easy to be in the excitation phase. This result leads to the phenomenon of both ends of the slow path conduction to the middle, when the 2 directions of excitation meet, because the forward cardiomyocytes are entering the absolute response period, so the excitation is canceled, this phenomenon is also called active inhibition, the fast path in the atrioventricular node conduction is in an absolutely strong position, and eventually form a normal phenomenon of 1:1 AV conduction. This is why almost everyone has a double pathway but the vast majority of people do not develop the disease. In some pathological states, such as congenital dysplasia, the active inhibition phenomenon disappears, and the excitation retarding path passes down to the bundle and subsequent pathways, which can trigger tachycardia, manifested as 1:2 conduction between AV (Fig. 9). In this case, a review of the surface electrocardiogram of the out-of-hospital attack found that there was a regular P:QRS = 1:2 performance in the V1 lead (Fig. 10). In addition, studies have found that DAVNNT has 6 atypical ECG modes in addition to the typical ECG mode of fast and slow path 1:2 AV conduction.^[10–16]^ It includes anterograde block with fast and slow path, fast and slow path, alternate path block, functional bundle branch block, and 1:2 AV block with AVNRT.^[10–16]^ It has been reported that a single case can be combined with multiple block phenomena^[17]^ (Fig. 5), and more findings may be made in this disease, such as improving the Holter electrocardiogram before surgery.

The difference between atrioventricular nodal reentrant tachycardia and atrioventricular nodal reentrant tachycardia is that when the former excites the slow path to the bundle, the fast path has a long refractory period and has not yet recovered the excitable state, so the excitation can only pass down the bundle and cause 2 ventricular beats. When the latter excites the slow path to his bundle, the fast path has been restored to the excitation period, and the excitement will be extended to the upward and reverse transmission of the fast path and the downward transmission of the bundle respectively. When the reverse transmission reaches the fork of the fast path and the slow path at the head of the atrioventricular node, a return will be completed, so that the slow path will restore its excitability, and the excitement will continue to extend the downward transmission of the slow path. If the cardiomyocytes in the pathway can recover excitability upon the arrival of excitation, the excitation will continue to return in the atrioventricular node and induce ventricular excitation.^[18,19]^ Both conditions lead to increased ventricular beats and tachycardia, but the mechanisms are obviously different.

In addition to this, the case needs to be differentiated from sinus tachycardia, accelerated junctional escape beats, atrial prematurity/atrial tachycardia, atrial fibrillation, and ventricular prematurity/ventricular tachycardia by means of surface electrocardiograms, esophageal pacing,^[20]^ and intracardiac electrophysiologic examination. (1) Sinus tachycardia: Sinus tachycardia is generally a phenomenon of fast normal heart rate, and most of which have inducement, such as emotional excitement, drinking tea or coffee, fever, etc. The clinical manifestations of AV intervals are 1:1 conduction, and sinus P wave is present before the QRS wave of the surface electrocardiogram. (2) Accelerated nodule escape: This type of tachycardia is mostly pathological and rarely physiologic. The body surface electrocardiogram presents a narrow QRS wave group, and the tachycardia presents a warm and awake phenomenon, that is, the heart rate gradually increases and then gradually slows down when it terminates, often accompanied by atrioventricular interference disjunction.^[21–25]^ (3) Atrial premature/atrial tachycardia: QRS wave group of body surface electrocardiogram appears with ectopic P wave, which may be difficult to identify, and intracardiac electrophysiological examination can confirm the diagnosis. In general, the frequency of A-wave is significantly faster than that of V-wave, and AV can also be 1:1 conduction. (4) Atrial fibrillation: DAVNNT is the disease most likely to be misdiagnosed as atrial fibrillation.^[1,26–28]^ The P wave of body surface electrocardiogram disappears and is replaced by f wave. The RR interval is absolutely uneven, which is different from the regular arrhythmia of DAVNNT. (5) Ventricular premature/ventricular tachycardia: Ventricular QRS has a large abnormal shape, which is obviously different from sinus QRS, accompanied by atrioventricular separation, fusion wave and other characteristic phenomena. In the electrophysiological examination of the heart, the frequency of V-wave is obviously faster than that of A-wave, which can be identified according to the relationship between atrial and ventricular signals. In this case, a large abnormal QRS wave was occasionally seen, because there were no P waves before and after all wide QRS waves. Moreover, there is a clear proportional conduction relationship between AV, so it is considered as functional bundle branch block, which is not consistent with this arrhythmia.^[23,25–27,29–35]^

Because DAVNNT causes tachycardia, it can also cause palpitations, tachycardia cardiomyopathy,^[7,11–13,15,31,35–37]^ heart failure, implantable cardioverter defibrillator discharge,^[27,29]^ etc. Once found, it needs to be treated as soon as possible, and its treatment is similar to that of AVNRT. The existence of redundant pathways in the atrioventricular node is a key link in its pathogenesis, and the removal of the redundant pathways is the key to treatment.^[7,16,25]^ Modified ablation of the slow pathway of the atrioventricular node is currently an effective treatment for DAVNNT, and experienced central ablation has an immediate success rate of over 95%, with complications similar to those of AVNRT. The incidence of complete atrioventricular block is <1%.^[27,38]^

## 4. Limitation

This case is a special value discovered during the review of previous surgical cases. Although the integrity of the current case is still acceptable, there may be some details forgotten due to the long history. This is also a profound lesson for us. If we encounter valuable cases in the future, we will promptly organize them in order to preserve the absolute integrity of the case. Due to some reasons, the failure to place HRA electrodes during the surgery did not affect the surgical outcome, but it also made the surgery lose its standardization. We will also keep this lesson in mind. During the follow-up process, due to living in rural areas, the patient did not arrive at the hospital 3 months after discharge. We only learned through phone contact that she did not have symptoms such as palpitations or tachycardia, as there was no evidence of ECG or dynamic electrocardiogram left, which is very regrettable.

## Acknowledgments

This study was supported by a scientific research project from the National Natural Science Foundation of China (Grant Nos. 82200316, 82160057, and 81860061). Guizhou Province Science and Technology Plan Project (ZK [2021]353). This study was also supported by the Science and Technology Fund of the Guizhou Provincial Health and Health Commission (gzwkj2023-133).

## Author contributions

**Methodology:** Lei Chen.

**Writing – original draft:** Yixuan Gao.

**Writing – review & editing:** Yixuan Gao, Yan Wang, Ranzun Zhao, Du Yang, Lei Chen, Bei Shi.
